# Investigating the Effect of Cigarette Smoking on Serum Uric Acid Levels in Multiple Sclerosis Patients: A Cross Sectional Study

**DOI:** 10.3390/brainsci13050800

**Published:** 2023-05-15

**Authors:** Mohammed Alrouji, Ali Manouchehrinia, Jehan Aram, Abdulmajeed Alotaibi, Sharif Alhajlah, Yasir Almuhanna, Othman Alomeir, Anas Shamsi, Bruno Gran, Cris S. Constantinescu

**Affiliations:** 1 Clinical Neurosciences Group, Shaqra University, Shaqra 11961, Saudi Arabia; 2 Division of Clinical Neuroscience, School of Medicine, University of Nottingham, Nottingham NG7 2UH, UK; 3 Department of Clinical Medical Laboratories, College of Applied Medical Sciences, Shaqra University, Sahqra 11961, Saudi Arabia; 4 Department of Clinical Neuroscience, Karolinska Institute, 171 77 Solna, Sweden; 5 College of Applied Medical Sciences, King Saud bin Abdulaziz University for Health Sciences, Riyadh 14611, Saudi Arabia; 6 Department of Clinical Laboratory Sciences, College of Applied Medical Sciences, Shaqra University, Shaqra 11961, Saudi Arabia; 7 Department of Pharmacy Practice, College of Pharmacy, Shaqra University, Shaqra 11961, Saudi Arabia; 8 Centre of Medical and Bio-Allied Health Sciences Research, Ajman University, Ajman 346, United Arab Emirates; 9 Department of Neurology, Nottingham University Hospitals NHS Trust, Nottingham NG7 2UH, UK; 10 Cooper University Hospital, Cooper Neurological Institute, Camden, NJ 08103, USA

**Keywords:** smoking, cigarette smoke, multiple sclerosis, neuroinflammation, oxidative stress, uric acid

## Abstract

Objectives: The present study is aimed at determining the effect of cigarette smoking (CS) on serum uric acid (UA) levels quantitatively before and after smoking cessation among people with MS (pwMS). Additionally, a possible correlation between UA levels and both disability progression and disease severity was also investigated. A retrospective cross-sectional study was conducted using the Nottingham University Hospitals MS Clinics database. It involves 127 people with definite MS recorded when reporting the latest smoking status and the clinical diagnosis. All necessary demographics and clinical characteristics were collected. We found that smoker pwMS had significantly lower serum UA levels than non-smoker pwMS (*p*-value = 0.0475), and this reduction was recovered after smoking cessation (*p*-value = 0.0216). However, the levels of disability or disease severity were not correlated with the levels of serum UA in current smoker pwMS, measured by the expanded disability status scale (EDSS; r = −0.24; *p*-value = 0.38), multiple sclerosis impact scale 29 (MSIS-29; r = 0.01; *p*-value = 0.97) and MS severity score (MSSS; r = −0.16; *p*-value = 0.58), respectively. Our result suggests that the reduction in UA levels is more likely a consequence of oxidative stress triggered by many risk factors, including CS, and could be considered a potential indicator of smoking cessation. In addition, the absence of a correlation between UA levels and disease severity and disability suggests that UA is not an optimal biomarker for disease severity and disability prediction among current smoker, ex-smoker or non-smoker pwMS.

## 1. Introduction

Accumulated data suggest a major role of oxidative stress in multiple sclerosis (MS) pathogenesis. Reactive oxygen species (ROS) and reactive nitrogen species (RNS) are primarily produced in excess amounts by activated microglial cells and brain-resident macrophages. They are mediators in MS and experimental autoimmune encephalomyelitis (EAE) pathogenesis [1,2,3]. ROS and RNS are more likely to be in a radical form, such as superoxide anion (radical of oxygen), nitric oxide (radical of nitrogen) and others. Nitric oxide is non-deleterious and less reactive, but it can form peroxynitrite (one of the most harmful ROS/RNS known) when reacting with superoxide anion [4]. Natural antioxidants, such as uric acid, superoxide dismutase and others, counterbalance those ROS and RNS.

In pathological conditions, the natural antioxidant defence levels will be overwhelmed by high levels of generated ROS/RNS and cause oxidative stress [5]. Furthermore, there is an agreement that the “final common molecule” for oxidative stress is peroxynitrite, which is formed due to nitric oxide production and interaction with ROS and is involved in protein, lipid and DNA oxidation as mitochondrial dysfunction [6,7,8,9]. Oxidized lipids and DNA were reported to be highly enriched in active MS lesions. They were associated with inflammation, determined by several CD3+ T cells, macrophages and microglia in the lesions [10]. In activated microglia, Fischer and colleagues found that molecules of the nicotinamide adenine dinucleotide phosphate (NADPH) oxidase (NOX) complexes such as transmembrane proteins p22phox, gp91phox and p47phox were constitutively expressed and upregulated, suggesting that the inflammation associated oxidative burst through ROS production by NADPH oxidases plays a crucial role in MS pathogenesis by driving both demyelination and neurodegeneration [11,12].

Uric acid (UA), the end product of purine catabolism and a powerful natural antioxidant, is a major part of human plasma’s total antioxidant scavenging activity [13]. The ability to scavenge peroxynitrite-derived radicals before they react with their targeted molecules and cause damage qualifies UA as an effective neuroprotectant [14,15].

Many observations provide convincing evidence for the effective role of UA in MS. Hooper and colleagues found that treatment of EAE with high doses of UA completely protected mice from the disease [16]. In addition, serum UA is reported to be significantly lower in people with MS [17,18,19,20,21]. Furthermore, it has been shown that during treatment with MS immunotherapies, an increased serum UA level was observed. Treatments included glatiramer acetate (Copaxone), interferon-beta (IFNβ) and high-dose methylprednisolone [22,23,24].

Despite the persuasive clinical and experimental data on higher serum UA importance to MS risk, in randomized control trials, oral administration of an inducer of inosine ≤ 10 mg/dl, a precursor of uric acid, maintained for up to two years in combination with IFNβ, as disease-modifying treatment, did not add any additional benefit to disability accumulation compared to IFNβ alone [25,26].

Cigarette smoking, the leading cause of morbidity worldwide, has been shown to play an important role in the aetiology of MS. Cigarette smoke constituents can contribute to the excessive or dysregulated generation of ROS. Among many potential sources of ROS, xanthine oxidase, a final enzyme of purine metabolism, and nicotinamide adenine dinucleotide phosphate (NADPH) oxidase (NOX) have been implicated [27,28,29]. Ko and colleagues reported that cigarette smoke extract (CSE) induces intracellular ROS in both human and mouse macrophages due to the activation of NADPH oxidase, which in turn activates AMP-activated protein kinase (AMPK)/MAPK signalling with NF-κB [30].

To date, investigations on the relationship between cigarette smoking and serum UA levels in people with MS are lacking; however, a few studies have addressed it in healthy volunteers, people with gout and preeclampsia syndrome, a condition that affects some pregnant women characterized by high blood pressure and other manifestations such as severe headache, nausea or vomiting, excessive weight gain and severe heartburn [31,32,33,34,35,36,37].

This study aimed to determine the effect of cigarette smoking on serum UA levels quantitatively before and after smoking cessation among people with MS and to further determine the possible correlation between serum UA levels and both disability progression and disease severity.

## 2. Results

Descriptive statistics for demographics and clinical characteristics were used to categorise the data of the patients included in this study (Table 1).

**Figure 1:**
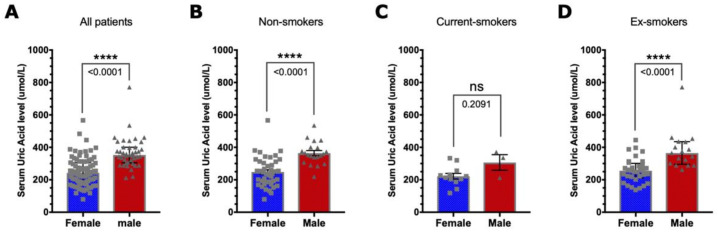
Difference in UA levels between men and women. As appropriate, the Mann–Whitney U test or Welch’s *t*-test was used. Bar graphs show the difference in UA among (**A**) all patients included in this study (**** *p*-value = 0.0001), (**B**) non-smokers (**** *p*-value = 0.0001), (**C**) current smokers (*p*-value = 0.209) and (**D**) ex-smokers (*p*-value = 0.0001). The mean with the SEM in (**B**,**C**) and the median with the interquartile range in (**A**,**D**) are indicated by bars.

Before comparing the effect of smoking on serum UA levels and the correlation between UA and disability and disease severity, the serum UA levels were compared between males and females among all study subjects and each category of smoking status. As shown in Figure 1, the serum UA level significantly differs between men and women in non-smokers and ex-smokers (*p*-value = 0.0001 and 0.0001, respectively), but it is not significant amongst current smokers (*p*-value = 0.21).

**Figure 2:**
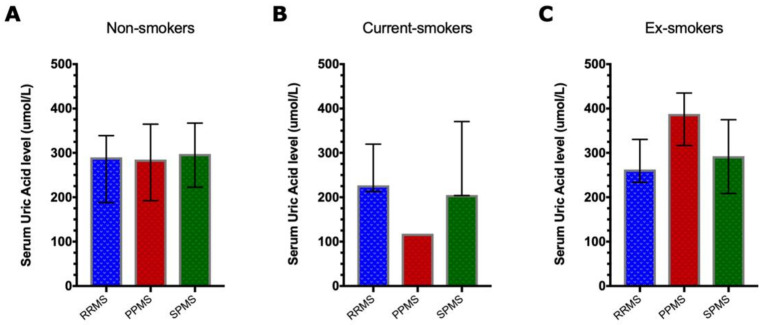
UA levels and MS clinical course. The bar graphs show the serum UA level variations according to the disease course in different smoking status categories. Using ordinary one-way ANOVA, no significant differences in UA were found between the disease course groups of non-smoker people with MS (*p*-value = 0.79) (**A**). The same applies to current smokers (*p*-value = 0.22) as shown in (**B**) and ex-smokers (*p*-value = 0.065) (**C**). The median with the interquartile range is indicated by bars.

Furthermore, each smoking category was sub-grouped based on the disease course, and then they were compared for the levels of UA. Among the category of non-smoker people with MS, no significant differences were found between people with RRMS, PPMS and SPMS diagnosis (*p*-value = 0.79). The same applies to current smokers and ex-smokers: (*p*-value = 0.22) and (*p*-value = 0.065), respectively (Figure 2).

**Figure 3:**
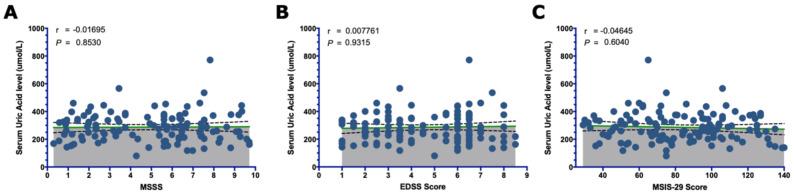
The overall correlation between serum UA levels and MS clinical characteristics. Using Spearman’s correlation test, no correlation between the MS severity score (MSSS) and UA was found (**A**). In addition, it does not correlate with disability (**B**,**C**). The mean with the 95% confidence interval (95% CI) is indicated in the dot blot graph by the green line and the dotted lines, respectively. (r = stands for the Spearman product-moment correlation coefficient).

Additionally, we studied the overall correlation between serum UA levels and the clinical measures associated with the disease severity and disability of all patients included in this study, including non-smokers, current smokers and ex-smokers. No association was found between UA level and disease severity (r = −0.02; *p*-value = 0.85) or the disability scores, measured by EDSS (r = 0.007; *p*-value = 0.93) and MSIS-29 (r = −0.05; *p*-value = 0.60) (Figure 3).

### 2.1. Reduction of Serum UA by Smoking

**Figure 4:**
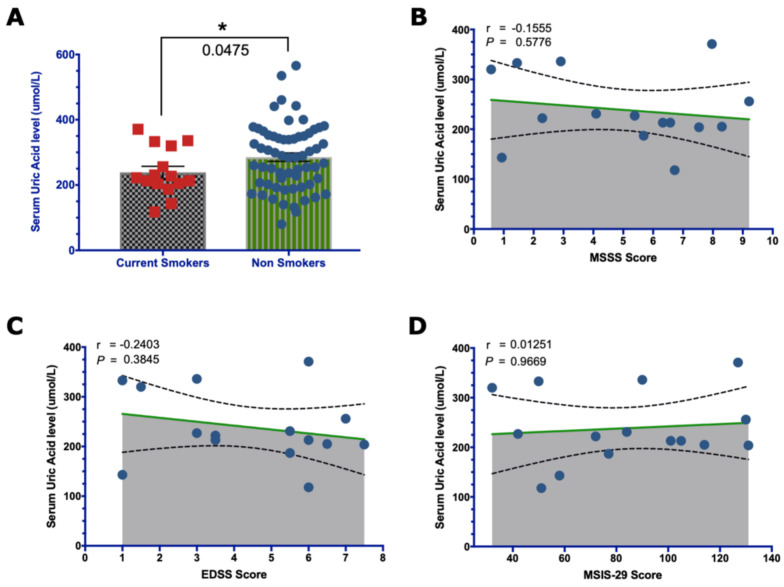
Effect of smoking on UA levels. (**A**) The bar graph shows lower UA levels in smokers than in non-smokers (two-tailed * *p*-value = 0.047; Welch’s *t*-test). The correlation analysis of Spearman’s correlation test shows that having a low serum UA level is not correlated with disease severity (**B**) or an increased disability score, measured using EDSS (**C**) and MSIS-29 (**D**). The means with the SEM of the UA levels are compared in (**A**), and the means with the 95% CI are shown in the graphs (**B**–**D**).

We found that serum UA was significantly lower in current smoker people with MS compared to non-smokers (*p*-value = 0.0475). Additionally, among current smokers, there was no correlation between having lower serum UA levels and having severe disease (r = −0.16; *p*-value = 0.58) and high disability scores, measured by EDSS (r = −0.24; *p*-value = 0.38) and MSIS-29 (r = 0.01; *p*-value = 0.97) (Figure 4).

### 2.2. Effect of Smoking Cessation on UA

**Figure 5:**
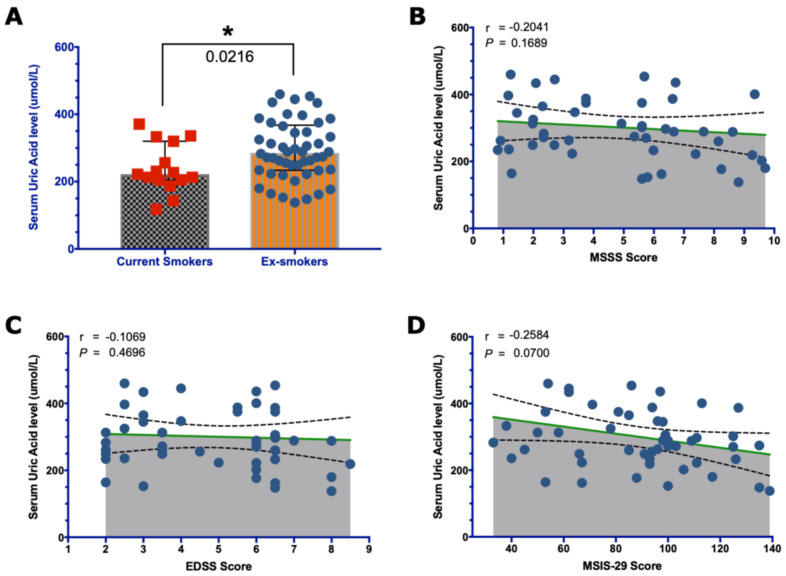
Effect of smoking cessation on serum UA levels. (**A**) The bar graph shows that quitting smoking significantly increases UA levels (two-tailed * *p*-value = 0.0216; Mann–Whitney U test). The Spearman’s correlation test shows the correlation between UA and both the disease severity (**B**) and disability scores, measured by EDSS (**C**) and MSIS-29 (**D**). The median with the IQ range of UA is compared in (**A**), and the means with the 95% CI are shown in graphs (**B**–**D**).

Among people with MS, the level of UA was significantly higher in ex-smokers than in current smokers (*p*-value = 0.0216) (Figure 5A), but it was not significantly higher when compared with non-smokers (*p*-value = 0.59; graph not shown). Moreover, there was no correlation found between higher serum UA levels and disease severity (r = −0.20; *p*-value = 0.17) and disability scores when measured by EDSS (r = −0.11; *p*-value = 0.47), but there was a trend towards a negative correlation when measured by MSIS-29 (r = −0.26; *p*-value = 0.07) (Figure 5B,D).

## 3. Discussion

To the best of our knowledge, this is the first study investigating the effect of cigarette smoking on UA in people with MS and its impact on disease severity and disability. As expected, based on previous reports where women are known to have lower serum UA, we observed lower levels of serum UA in women compared to men amongst all study subjects [17,18,19,20,21], and there was no difference in UA levels in MS patients when sub-grouped based on the clinical course of the disease: RRMS, PPMS or SPMS. Additionally, no overall correlation was found between UA level and MS severity or levels of disability. We also found that cigarette smoking reduces the levels of serum UA, and this reduction was recovered by smoking cessation. Furthermore, no correlations were found between serum UA level and MS clinical measures for disease severity and disability.

Lower UA levels in serum from people with MS, when compared with healthy controls or with people with other inflammatory neurological diseases, have been reported in many studies [17,18,19,20,21,38]. In addition, it has been shown that female MS patients have lower UA levels compared to male patients [18,39,40,41]. In line with what has been previously shown, we found that male MS patients had significantly higher serum UA levels than female patients (*p*-value = 0.0001; Figure 1A). It should be noted that levels of serum UA are variably affected in several neurological and non-neurological diseases [42,43]. Hence, its pathophysiological importance still has to be fully clarified in MS.

The studies were inconsistent regarding the correlation between UA levels and clinical measures such as disease activity, severity and disability. We found no overall correlation between UA levels and disease severity, measured by MSSS, or disability, measured by EDSS and MSIS-29, as shown in Figure 3. Additionally, no trend was observed toward lower levels of UA when compared between the three different MS disease courses (RRMS, PPMS and SPMS) among non-smoker patients, which is consistent with what has been reported by previous studies [18,19,43]. These results suggest that the role of UA in MS severity and disability is far from clear, and MS patients might have a primary antioxidant reserve deficiency. However, despite the uncertainty about whether low UA levels in MS are a cause or consequence of the disease, Knapp and colleagues found that patients with optic neuritis (ON) as a clinically isolated syndrome of MS had significantly low UA levels suggesting that a reduced antioxidant reserve might be an early pathogenic mechanism in inflammatory demyelination [41].

Chronic cigarette smoking, as one of the modifiable risk factors, has been shown to increase the risk of developing MS [44,45,46]. Additionally, its influence on disease and disability accumulation has been suggested. Cumulative evidence has shown that smoker people with MS experience more severe motor symptoms [47], have more T2 and gadolinium-enhancing lesions on MRI [48] and have increased psychological impairment [49,50] and increased conversion from clinically isolated syndrome (CIS) to clinically definite MS by about 80% [51]. Previous studies have reported the effect of smoking on increasing free radical production and overall antioxidant depletion, including UA, in healthy people [32,36,52]; however, this is still to be clarified in people with MS. In addition, it has been shown that even non-smokers exposed to cigarette smoke have a significantly lower antioxidant reserve than unexposed non-smokers [53], which is in line with the evidence showing that the risk of developing MS is influenced by passive smoking [54].

In the current study, we found that smoker MS patients had significantly lower serum UA levels than non-smoker patients, and this reduction was recovered after smoking cessation. The decrease in serum UA among current smoker people with MS can be partially explained by the increased activity of xanthine oxidoreductase (XOR) in response to cigarette smoke. This enzyme consists of inter-convertible forms, including xanthine dehydrogenase (XDH) and xanthine oxidase (XO), and plays a central role in purine catabolism by converting hypoxanthine to xanthine and xanthine to uric acid and/or ROS.

It has been reported that plasma XOR and XO activity is significantly higher in healthy smokers than in non-smokers [55,56]. Furthermore, the expression and activity of XOR were induced by cigarette smoke condensate (CSC) or extract (CSE), resulting in oxidative stress and cell apoptosis in cultured pulmonary endothelial cells [28,57]. Inhibiting XOR activity with allopurinol, XOR inhibitors used for gout treatment, reversed the cigarette smoke-induced endothelial dysfunction in healthy volunteers instantly, despite reducing serum UA levels [58]. Similarly, in cultured primary human lung microvascular endothelial cells (HLMVECs) and C57/bl6 mice, cigarette-smoke-induced XOR activity and protein levels were remarkably attenuated when treated with allopurinol or febuxostat; the latter is a non-purine-selective-XOR-inhibitor used for gout treatment [57].

Very little is known about the role of ROS-generating enzyme XOR in MS, its relationship with CNS autoimmunity and the effect of smoking on this enzyme. Recently and for the first time, Honorat and colleagues found, in the EAE mouse model of MS, that the activity of XO was increased in the serum and within the CNS of the mice with experimental inflammatory demyelination, and the administration of febuxostat was able to reduce the clinical signs of the disease, suppress the excess ROS production from infiltrating macrophages and microglia, improve mitochondrial function and prevent neurodegeneration in both relapsing–remitting and secondary progressive EAE [59,60]. Our current study found that serum UA levels did not influence the clinical measures for disease severity and disability among non-smoker, current smoker or ex-smoker people with MS, which is in line with what has been reported among non-smoker MS patients only [18,19,39,43,61]. Therefore, we suggest that the role of UA in disease severity or disability is complex and incompletely elucidated. UA might not be a primary deficit and potential biomarker for disease severity or disability, as suggested recently [38]. Still, it could be a marker for ongoing oxidative stress induced by ROS generation through inflammation and/or cigarette smoking, which leads to endothelial loss of function and integrity of the blood–brain barrier (BBB), increasing the infiltration of inflammatory cells into the CNS, as several studies have reported it. In addition, an increase in UA levels could be a helpful biomarker that can be correlated to better biological and clinical outcomes. In the EAE mouse model, for instance, UA administration was shown to enhance the permeability of the BBB and interfere with the inflammatory cell invasion into the CNS [62,63]. In contrast, the oxidative capacity and pro-inflammatory activity of cigarette smoking compromised the function, integrity and damage of the BBB [64,65,66,67].

Of note, UA is not the only antioxidant mechanism detoxing reactive oxidants. It has been reported that other antioxidant enzymes, such as superoxide dismutase (SOD), catalase and glutathione peroxidase (GPx), are compromised in people with neurological diseases, including MS, suggesting their importance in protection against ROS within the brain. ROS sources in MS are mainly from mitochondria and ROS-producing enzymes such as nicotinamide adenine dinucleotide phosphate (NADPH) oxidases (NOXs), myeloperoxidase (MPO) and inducible nitric oxide (NO) synthase (iNOS), which is upregulated in the active stage of MS [68]. Nuclear-factor-erythroid-2-related factor 2 (Nrf2) controls the expression of most antioxidant enzymes through its binding to the antioxidant response element in the promoters of target genes, such as heme oxygenase-1, NADPH: quinone oxidoreductase-1, peroxiredoxins and several proteins involved in glutathione (GSH) metabolism [69,70,71].

Despite being the first study to investigate the relationship between smoking and UA levels and its correlation with disease severity and disability in MS directly, this study has some limitations. Firstly, a larger sample size with matched age, sex and MS clinical course to estimate the effect of smoking on UA would be more convincing. Secondly, for an accurate interpretation, confounding variables that possibly modify UA levels, such as BMI, alcohol intake, drugs, number of cigarettes smoked/day, comorbidities and disease activity, using neuroradiological assessment, should be considered to enable the use of more powerful statistical methods and reduce the potential effect of these confounding factors. Finally, information about XOR activity, mitochondrial dysfunction and ATP consumption would be useful.

## 4. Materials and Methods

### 4.1. Setting and Participants

The retrospective study population included people with definite MS registered in Nottingham University Hospitals MS Clinics database, a major regional referral centre for MS in England’s East Midlands counties. All of the clinically definite MS diagnoses were made by an MS specialist neurologist, according to the McDonald and/or Poser criteria [72], and were supported by a positive magnetic resonance imaging (MRI) scan. Of all the patients registered in the clinic, a database of around 1032 patients was created by one MS consultant neurologist (C.S.C.) and was used in prospective follow-up studies [73,74,75]. The patients are routinely followed up and undergo neurological and medical evaluation, which is systematically documented with the reporting history, investigation results, clinical disease course, disability score and treatments. Of these, we obtained the laboratory results of serum UA levels for 127 people with a definite MS diagnosis recorded when reporting the latest smoking status and the clinical diagnosis. The study was approved by the National Research Ethics Service (NRES) East Midlands Ethics Committee Derby-1.

### 4.2. Smoking History and Clinical Data

The patients’ smoking status history was obtained during their first visit to the clinic at the time of disease onset or diagnosis. It was updated and recorded more than once during the regular clinical follow-ups, and the latest recorded smoking status was used. The patients were categorised based on their smoking status as non-smokers, current smokers or ex-smokers. The age, sex, disease duration, latest expanded disability status scale (EDSS) score [76] and serum UA recorded were used. The level of physical and psychological disability was measured by the Multiple Sclerosis Impact Scale 29 (MSIS-29), reported previously [74] through a 29-question patient-reported questionnaire [77]. Furthermore, the MS severity score (MSSS) was also calculated and used in this report by integrating the disease duration and EDSS score according to the guidelines published by Roxburgh [78]. The course of MS is referred to as relapsing–remitting (RRMS), primary progressive (PPMS) or secondary progressive (SPMS).

### 4.3. Statistical Analysis

Normality was tested using both Shapiro–Wilk and D’Agostino and Pearson tests. Where appropriate, differences between mean values were evaluated by the parametric Welch’s *t*-test, ordinary one-way ANOVA for normally distributed data and the non-parametric Mann–Whitney U test for non-normally distributed data. To test the correlation between UA levels and both disability progression and disease severity, Pearson’s or Spearman’s product-moment correlation coefficient (r) test was used as appropriate unless otherwise stated. Two-tailed *p*-values of 0.05 were considered to indicate statistical significance.

## 5. Limitations

Despite being the first study to directly investigate the relationship between smoking and UA levels and its correlation with disease severity and disability in MS, there are some limitations to this study. Firstly, a larger sample size with matched age, sex and MS clinical course to estimate the effect of smoking on UA would be more convincing. Secondly, for an accurate interpretation, confounding variables that possibly modify UA levels, such as BMI, alcohol intake, drugs, number of cigarettes smoked/day, comorbidities and disease activity, using neuroradiological assessment, should be considered to enable the use of more powerful statistical methods and reduce the potential effect of these confounding factors. Finally, information about XOR activity, mitochondrial dysfunction and ATP consumption would be very useful.

## 6. Conclusions

Considering our results and the literature, we suggest that the reduction in UA levels is more likely a consequence of oxidative stress triggered by many risk factors such as infection, smoking and others preceding disease onset or during the clinical course of the disease. Furthermore, the absence of a correlation between UA levels and disease severity and disability among non-smoker, current smoker or ex-smoker MS patients suggests that UA levels are not a good biomarker for disease severity and/or disability progression. However, it could be recommended as a routine clinical test since it is a simple and cheap indicator that reflects part of the oxidative stress status. In addition, the increase in UA in ex-smokers could allow longitudinal monitoring after smoking cessation.

Further prospective studies are needed, considering the above limitations, to evaluate the effect of smoking on serum UA variations and XOR activity during the MS course and the possible relationships with the clinical features of MS including clinical relapses, disease severity and disability progression. In addition, future studies should now explore whether the XOR inhibitors allopurinol or febuxostat will, in practice, at least as adjuvant strategies, reduce the risk of conversion from CIS to definite MS, the number of relapses and disability progression since people with gout very rarely develop MS which we think could be due to the effect of the treatment rather than the elevated UA levels. Despite the possible reduction in UA levels after treatment with XOR inhibitors, the inhibition of XOR may be more important than the reduction in the assumed protection by UA, possibly because peroxynitrite production is reduced. Thus, there is less need for it to be scavenged by UA.

## Figures and Tables

**Table 1:** Demographics and clinical characteristics.

Characteristics	Smoking Status		
	Non-Smokers (*n* = 62)	Current Smokers (*n* = 15)	ExSmokers (*n* = 50)
Age (mean, SD *)	52.87 (±13.38)	46 (±9.33)	54.56 (±8.70)
Sex (female)	42 (67.74%)	12 (80%)	33 (66%)
Serum Uric Acid (mean, SD; umol/L)	285.5 (±100)	238.6 (±72.36)	300.7 (±108.80)
EDSS (median, IQR *)	5 (±3)	5.5 (±3)	5.75 (±3.5)
MSIS-29 (mean, SD)	77.4 (±27.68)	84.27 (±33.19)	88.36 (±27.35)
MSSS (mean, SD)	4.80 (±2.62)	5.06 (±2.84)	4.87 (±2.75)
Course of the disease			
Relapsing–remitting (RRMS)	35 (56.45%)	11 (73.33%)	26 (52%)
Primary progressive (PPMS)	07 (11.29%)	01 (6.66%)	05 (10%)
Secondary progressive (SPMS)	20 (32.25%)	03 (20%)	17 (34%)

* (SD: standard deviation; IQR: interquartile range).

## Data Availability

All the associated Data is contained within the article.
